# Particulate Constituents and Posttransplant Outcomes Among Kidney Transplant Recipients

**DOI:** 10.1001/jamanetworkopen.2025.27142

**Published:** 2025-08-14

**Authors:** Yijing Feng, Yiting Li, Sunjae Bae, Babak Orandi, Mara McAdams-Demarco, Joel Schwartz

**Affiliations:** 1 Department of Epidemiology, Harvard T.H. Chan School of Public Health, Boston, Massachusetts; 2 Department of Surgery, New York University Grossman School of Medicine, New York; 3 Department of Population Health, New York University Grossman School of Medicine, New York; 4 Department of Medicine, New York University Grossman School of Medicine, New York; 5 Department of Environmental Health, Harvard T.H. Chan School of Public Health, Boston, Massachusetts

## Abstract

**Importance:**

Total particulate matter with a diameter of 2.5 µm or less (PM_2.5_) has been found to be associated with adverse posttransplant outcomes among kidney transplant (KT) recipients. However, PM_2.5_ is a complex mixture of multiple constituents, all of which have different toxicity profiles, so it is not clear which constituents are most associated with adverse outcomes among KT recipients.

**Objective:**

To investigate the associations between PM_2.5_ constituents and post-KT outcomes among KT recipients.

**Design, Setting, and Participants:**

This cohort study was conducted among patients who received a KT between January 2000 and December 2016 and lived in the contiguous United States. Follow-up continued through December 2021, and data were analyzed from August 2023 to May 2025.

**Exposures:**

Fifteen PM_2.5_ constituents (including elemental carbon, ammonium, nitrate, organic carbon [OC], sulfate [SO_4_^2-^], bromine, calcium, copper, iron, potassium, nickel [Ni], lead [Pb], silicon, vanadium, and zinc) at the zip code of residence prior to KT, estimated from ensembled machine learning models.

**Main Outcomes and Measures:**

Adverse post-KT outcomes included acute rejection, delayed graft function (DGF), death-censored graft failure (DCGF), and mortality. The association of PM_2.5_ constituents and the outcome were evaluated with weighted quantile sum regressions.

**Results:**

In total, 192 587 KT recipients were included in the analysis (mean [SD] age at transplant, 51.56 [13.47] years; 75 021 [39.0%] female; 51 455 [26.7%] Black, 28 586 [14.8%] Hispanic, and 97 927 [50.8%] White). Each decile increase in the PM_2.5_ constituent mixture was associated with a 6.8% (95% CI, 5.8%-7.8%) and 3.6% (95% CI, 2.1%-5.1%) increase in the odds of DGF and acute rejection, respectively. OC and Ni contributed the largest weights to the observed association between PM_2.5_ mixture and DGF (OC: relative importance, 35.6%; Ni: relative importance, 34.4%), while Pb had the largest impact on acute rejection (relative importance, 75.0%). Each decile increase in PM_2.5_ constituent mixture was associated with a 4.7% (95% CI, 3.3%-6.3%) and 3.9% (95% CI, 2.5%-5.2%) increase in the hazard of DCGF and all-cause mortality, respectively. The constituent that contributed the largest weight to the observed association between PM_2.5_ mixture and long-term post-KT outcomes was SO_4_^2-^ (relative importance, 51.3%).

**Conclusions and Relevance:**

In this cohort study, PM_2.5_ constituents were associated with an increased risk of adverse posttransplant outcomes among KT recipients. Of the PM_2.5_ constituents included in this study, SO_4_^2- ^contributed most to long-term outcomes, while Pb, OC, and Ni were more associated with short-term outcomes.

## Key Points

**Question:**

Are constituents of particulate matter with a diameter of 2.5 µm or less (PM_2.5_) associated with adverse posttransplant outcomes among kidney transplant recipients?

**Findings:**

In this cohort study with 192 587 kidney transplant recipients, exposure to PM_2.5_ mixture was associated with increased risk of delayed graft function, acute rejection, death-censored graft failure, and mortality after transplant. Of all the constituents studied, sulfate, lead, organic carbon, and nickel contributed the largest weights in the observed associations.

**Meaning:**

In this study, exposure to PM_2.5_ and its constituents, particularly sulfate, lead, organic carbon, and nickel, was associated with higher risks of adverse outcomes among kidney transplant recipients.

## Teaser

This cohort study evaluates whether the constituents of particulate matter are associated with adverse posttransplant outcomes among patients who received kidneys in the United States.

## Introduction

Particulate matter with a diameter of 2.5 µm or less (PM_2.5_) is a risk factor for kidney health. Previous epidemiology studies have suggested that PM_2.5_ is associated with increased risk of chronic kidney disease and kidney function decline.^1^ Kidney transplant (KT) recipients are likely to be vulnerable to the harmful effect of PM_2.5_. Previous studies suggested that PM_2.5_ has potential immunotoxic effects.^2^ Because of the chronic immunosuppression medications necessary for successful KT, alterations in the immune system may render recipients particularly susceptible to the infections and malignant neoplasms induced by air pollution.^3,4^ Moreover, PM_2.5_ could increase oxidative stress and systematic inflammatory responses,^2^ which are risk factors for adverse post-KT outcomes.

Emerging evidence suggests that PM_2.5_ is a risk factor for adverse posttransplant outcomes among KT recipients.^5,6^ However, these studies have generally treated PM_2.5_ as a single, uniform exposure, assuming that its harmful effects are the same across regions with similar total PM_2.5_ levels. In reality, PM_2.5_ is a complex mixture of constituents with varying toxicity profiles and health impacts.^7^ These constituents are emitted from different sources, which also vary geographically,^8^ meaning that even areas with identical total PM_2.5_ levels may experience different health effects due to differences in composition. The impact of individual PM_2.5_ constituents on post-KT outcomes remains unclear. Identifying the most harmful constituents could guide air pollution control strategies that specifically target these constituents or their primary sources. Such targeted approaches are likely to be more effective in reducing the adverse health impacts of PM_2.5_ compared with broad strategies aimed at reducing total PM_2.5_ levels. Moreover, the results from this study could also provide important information for KT recipients and candidates who seek to optimize their outcomes by living in less polluted areas or installing high-efficiency particulate arresting (HEPA) filters. Therefore, understanding how PM_2.5_ constituents affect post-KT outcomes is critical for improving the health of KT recipients. In this study, we aimed to evaluate the associations of particulate constituents with adverse post-KT outcomes, including delayed graft function (DGF), acute rejection, all-cause mortality, and death-censored graft failure (DCGF).

## Methods

This cohort study was approved by the institutional review board at New York University Grossman School of Medicine. It was determined exempt from institutional review board oversight and the requirement for informed consent under institutional policy, as the study participants could not be identified. We followed the Strengthening the Reporting of Observational Studies in Epidemiology (STROBE) reporting guideline for cohort studies.

### Data Source

This study used data from the Scientific Registry of Transplant Recipients (SRTR). The SRTR data system includes data on all donors, wait-listed candidates, and transplant recipients in the United States, submitted by the members of the Organ Procurement and Transplantation Network (OPTN). The Health Resources and Services Administration, US Department of Health and Human Services provides oversight to the activities of the OPTN and SRTR contractors. This dataset has previously been described elsewhere.^9^

### Study Population

We utilized SRTR data to identify adults who received their first KT between January 2001 and December 2016. To ensure consistency, we limited our analysis to participants with a residential zip code within the contiguous United States, excluding those with missing demographic, educational, recipient, or donor data. Follow-up started from the transplant date and ended on the date of loss to follow-up, death, or 5 years after transplant (until December 2021), whichever occurred first.

### Exposure Data

Mean annual levels of 15 PM_2.5_ constituents (including elemental carbon, ammonium (NH_4_^+^), nitrate, organic carbon [OC], sulfate [SO_4_^2-^], bromine, calcium, copper, iron, potassium, nickel [Ni], lead [Pb], silicon, vanadium, and zinc) were estimated at a resolution of 50 m × 50 m in urban areas and 1 km × 1 km in rural areas.^10,11^ The concentrations of the 15 PM_2.5_ constituents were estimated using advanced ensemble machine learning methods. These models integrated predictions from various machine learning algorithms and were then combined using either a geographically weighted generalized additive model or super-learning. Predictive models for PM_2.5_ constituents were developed using information from numerous sources, including satellite data, meteorological information, and land use factors. The cross-validated *R*^2^ values ranged from 0.80 to 0.96 for different constituents, which indicated strong model performance. Subsequently, the PM_2.5_ constituent concentrations at the grid cell level were aggregated to the zip code level.^12^ These environmental exposures were then linked to the participants based on their year of KT and residential zip codes.

The exposure in this study was defined as the moving average of PM constituents during the 1-year period prior to KT. If a recipient received KT in July of 2006, then the exposure would be calculated as

([7/12] × PM Component Level in 2006) + ([5/12] × PM Component Level in 2005).

### Outcome

The outcomes of this study were post-KT outcomes, including DGF, acute rejection, graft failure, and all-cause mortality. DGF was defined as the need for dialysis during the first week after KT. Acute rejection was defined as having any acute rejections episodes within the first year after transplantation. Graft failure was defined as returning to dialysis or transplant. Information on mortality in SRTR was obtained from multiple sources, including the National Technical Information Service’s Death Master File, the End-Stage Kidney Disease (ESKD) Death Notification Form from the Centers For Medicare& Medicaid Services, and the follow-up reports from transplant programs.^9^

### Other Covariates

In this study, we included individual factors and contextual factors as covariates. Individual level factors included age at transplant, sex, race and ethnicity (American Indian or Alaska Native, Arab or Middle Eastern, Asian, Black, Hispanic, Indian subcontinent, multiracial, Native Hawaiian or Other Pacific Islander, White, and unknown), education (high school graduate or greater and less than high school), body mass index (BMI), cause of ESKD (diabetes, hypertension, glomerulonephritis, and other), insurance type (public, private, and other), years receiving dialysis, donor type (deceased and living), and cold ischemia time (CIT). Race and ethnicity were collapsed into 4 categories: Black, Hispanic, White, and additional groups, which included American Indian or Alaska Native, Arab or Middle Eastern, Asian, Indian subcontinent, multiracial, and Native Hawaiian or Other Pacific Islander individuals as well as those with unknown race and ethnicity. The additional groups categories was created because of the small sample size of the racial groups included in it, which would limit the power of the analysis. We included race and ethnicity because they are important confounders and potentially effect measure modifiers in the exposure-outcome association. Individual factors and donor factors were obtained from SRTR. Contextual factors included median household income, percentage of population who lived in poverty, percentage of residents who were Black, percentage of Medicare beneficiaries who had at least 1 ambulatory visit in a year, and annual mean temperature at the zip code of residence. Contextual variables were obtained from the American Community Survey,^13^ the Dartmouth Atlas of Healthcare website,^14^ and the DAYMET database.^15^ The year of KT was also included as a covariate to account for potential temporal trends in both the particulate constituent levels and the practice of kidney care and KT.

### Statistical Analysis

We used weighted quantile sum regressions to evaluate the association between PM constituent mixtures and post-KT outcomes. Weighted quantile sum regression is a statistical method for estimating the effects of a mixture with correlated constituents; it assumes each quantile increase in the mixture index is linearly and unidirectionally associated with the outcome.^16^ The method allowed us to estimate the overall outcome associated with the mixture and the relative contribution of each constituent to those associations while accounting for the correlation between different constituents. The regression process was separated into 2 stages. Data were first separated into a test set and a validation set. In the first stage, constituents were transformed into quantiles (we chose deciles), and the relative importance of each constituent was estimated in the test set. To ensure robustness in estimating the weights (relative importance) for individual constituents, 100 bootstrap samples were taken of the training set, and the weights were fit in each sample. The mean weight for each constituent was then used in the second stage. All the weights for each constituent in the mixture were constrained to be positive and sum to 1. In the second stage, a mixture index was calculated by ∑^j^_i_*w*_i_*d*_i_ , where *w_i_* was the relative importance of the *i*^th^ constituent estimated from the first stage and *d_i_* was the level (in quantiles) of the *i*^th^ constituent. Then the outcomes associated with the mixture index was evaluated using a generalized linear model regression model in the validation set. This result estimates the outcome when the mixture index increased by 1 decile (ie, the overall association when each of the constituents increased by 1 decile).

DGF and acute rejections were binary outcomes; therefore, we used a weighted quantile sum regression with a logit link to investigate the exposure-outcome associations. In our primary analysis for graft failure, people who died before having an event was censored at the time of death. For the 2 time-to-event outcomes (graft failure and all-cause mortality), we used the pooled logistic regression model, which has been shown to approximate a Cox model, to evaluate the exposure-outcome associations. For each participant, we separated the follow-up period by months and created a new variable that indicates the number of months since transplantation. For example, if a participant were censored at 24 months after KT, they would have 24 observations in the analysis and be excluded from the risk set after 24 months. In our primary analysis, we assumed noninformative censoring. This model was used:

logit(*p*[*Y*_t_ = 0 | *Y*_t − 1_ = 0]) = β_0_ + β_1_ × *wqs* + β_z_′*Z* + β_t_′Splines(*t*),

where* wqs* indicates the mixture index, *Z* indicates the confounders, and *t* indicates months since transplantation (time variable). To allow for a flexible baseline hazard, we included a natural spline for the time variable. To identify vulnerable subgroups, we evaluated the effect measure modification by age at transplant (<55 years and ≥55 years), sex, race (White and all other racial and ethnic groups), donor type (deceased and living) and CIT (≤12 hours and >12 hours) by including the interaction term into the *wqs* model.

We conducted 3 sensitivity analysis. First, we restricted the analysis for DCGF and mortality to the first year after KT. Second, we further controlled for NO_2_ and ozone in the model. Finally, we ran a Cox proportional hazard model and another Fine and Gray competing risk model with total PM_2.5_ as the exposure to evaluate whether that competing risk had a large impact on the observed association between the exposure and graft failure.

All of the regression models were adjusted for recipient factors, donor factors, and contextual variables. The analysis was conducted using R version 4.1.3 (R Project for Statistical Computing) and Stata version 17 (StataCorp). Statistical significance was set at *P* < .05, and all tests were 2-tailed. Data analysis was conducted from August 2023 to May 2025.

## Results

In total, 192 587 KT recipients were included in the analysis (mean [SD] age at transplant, 51.56 [13.47] years; 75 021 [39.0%] female; 51 455 [26.7%] Black, 28 586 [14.8%] Hispanic, and 97 927 [50.8%] White). Of all the included KT recipients, 16 187 (8.4%) experienced acute rejection, 35 793 (18.6%) had DGF, 18 969 (9.7%) developed graft failure, and 21 750 (11.3%) died during the follow-up. Detailed baseline characteristics are shown in Table 1.

**Table 1.** Baseline Characteristics of the 192 587 Kidney Transplant Recipients Who Received Transplant in the United States Between 2001 and 2016

Characteristic	Recipients, No. (%) (N = 192 587)
Age at transplant, mean (SD), y	51.56 (13.47)
Sex	
Female	75 021 (39.0)
Male	117 566 (61.0)
Race and ethnicity	
Black	51 455 (26.7)
Hispanic or Latino	28 586 (14.8)
White	97 927 (50.8)
Additional groups^a^	14 619 (7.6)
BMI, mean (SD)	27.91 (5.50)
Cause of ESKD	
Diabetes	68 556 (35.6)
Hypertension	42 985 (22.3)
Glomerulonephritis	39 994 (20.8)
Other	41 052 (21.3)
Education level <high school degree	87 261 (45.3)
Insurance	
Public	121 871 (63.3)
Private	70 571 (36.6)
Other	145 (0.1)
Time receiving dialysis, mean (SD), y	2.91 (3.08)
Deceased donor	117 145 (60.8)
Cold ischemia time, mean (SD), h	12.84 (10.79)
Median household income, mean (SD), $	52 595.30 (21383.19)
Zip code–level factors	
<Poverty line, mean (SD), %	14.70 (9.09)
Black residents, mean (SD), %	17.04 (23.19)
Medicare beneficiaries with ≥1 ambulatory visit, mean (SD), %	76.81 (6.71)

Abbreviations: BMI, body mass index (calculated as weight in kilograms divided by height in meters squared); ESKD, end-stage kidney disease.

^a^ Additional groups included American Indian or Alaska Native, Arab or Middle Eastern, Asian, Indian subcontinent, multiracial, and Native Hawaiian or Other Pacific Islander individuals as well as those with unknown race and ethnicity.

Detailed distributions of the exposures are shown in Table 2. The correlations between particulate constituents are shown in the eFigure in Supplement 1.

**Table 2.** Zip Code Level PM_2.5_ Constituent Concentration During the Year Prior to Transplant Among the 192 587 Kidney Transplant Recipients Who Received Transplant in the United States Between 2001 and 2016

Constituent	Level, median (IQR)
Total PM_2.5_, µg/m^3^	9.9 (8.2-11.9)
Br, ng/m^3^	3.0 (2.6-3.4)
Ca, ng/m^3^	40.1 (30.1-58.2)
Cu, ng/m^3^	2.7 (1.7- 4.0)
EC, µg/m^3^	0.6 (0.4-0.7)
Fe, ng/m^3^	65.8 (50.7-84.7)
K, ng/m^3^	58.5 (51.9-67.2)
NH_4_^+^, µg/m^3^	0.9 (0.6-1.3)
Ni, ng/m^3^	0.6 (0.4-0.9)
NO_3_, µg/m^3^	1.1 (0.7-1.7)
OC, µg/m^3^	1.8 (1.5-2.3)
Pb, ng/m^3^	1.9 (1.3-2.9)
Si, ng/m^3^	85.4 (61.7-129.4)
SO_4_^2-^, µg/m^3^	2.1 (1.5-3.1)
V, ng/m^3^	0.8 (0.4-1.5)
Zn, ng/m^3^	8.1 (5.9-10.9)

Abbreviations: Br, bromine; Ca, calcium; Cu, copper; EC, elemental carbon; Fe, iron; K, potassium; NH_4_^+^, ammonium; Ni, nickel; NO_3_, nitrate; OC, organic carbon; Pb, lead; PM_2.5_, particulate matter with a diameter of 2.5 µm or less; Si, silicon; SO_4_^2-^ sulfate; V, vanadium; Zn, zinc.

After adjusting for the potential confounders, each 1-decile increase in the mixture of particulate constituents was associated with a 6.8% (95% CI, 5.8%-7.8%) increase in the odds of DGF among KT recipients. The particulate constituents with the highest relative importance were OC (35.6%), Ni (34.4%), and NO_3_ (21.3%).

Each 1-decile increase in the mixture of particulate constituents was associated with a 3.6% (95% CI, 2.1%-5.1%) increase in the odds of acute rejections. Overall, 75.0% was contributed by Pb (Figure).

**Figure.**
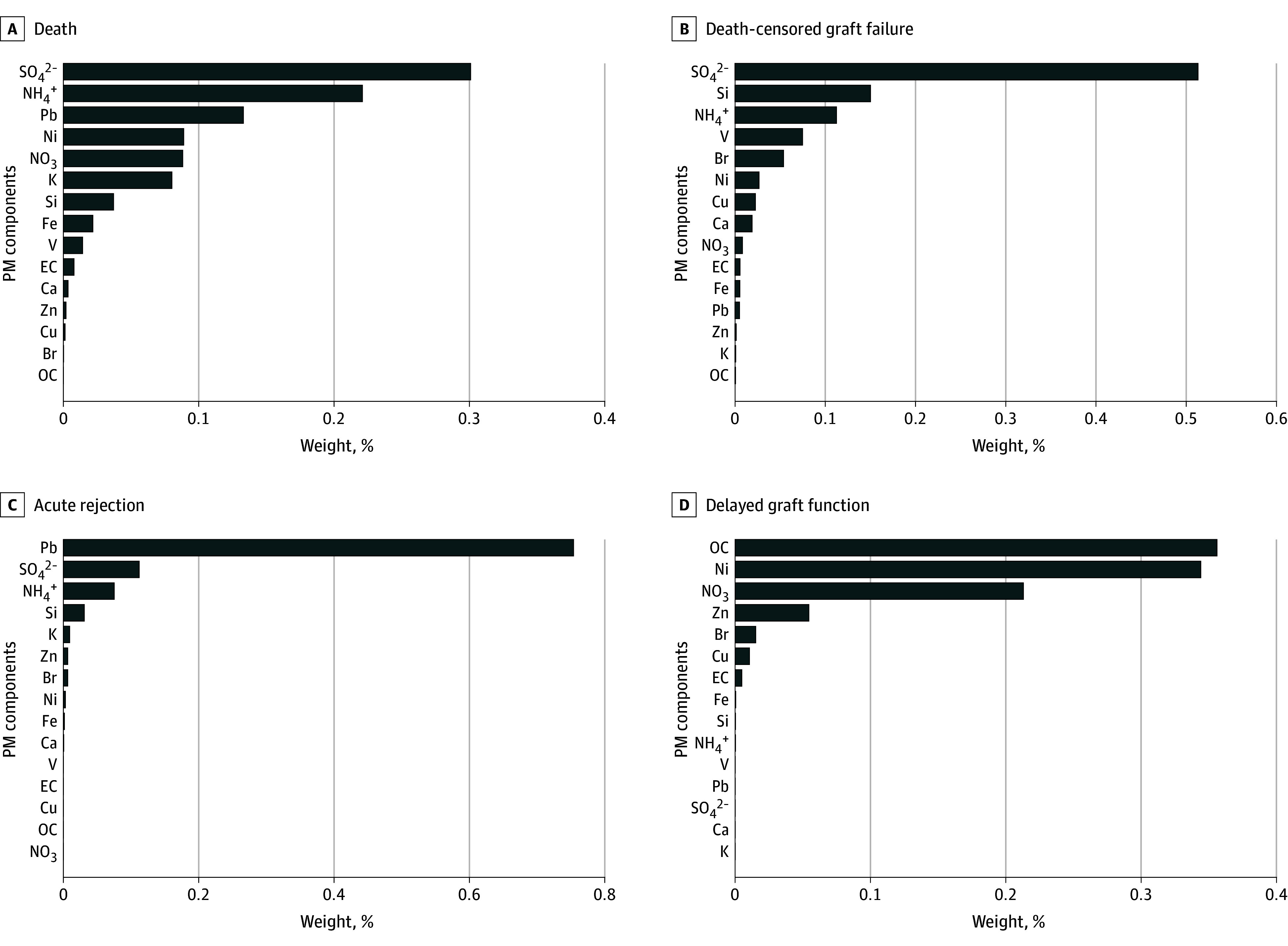
Estimated Contribution of Each Particulate Matter (PM) Constituent in the Association Between PM Mixture and Posttransplant Outcomes Among Kidney Transplant Recipients Br indicates bromine; Ca, calcium; Cu, copper; EC, elemental carbon; Fe, iron; K, potassium; NH4^+^, ammonium; Ni, nickel; NO_3_,^-^nitrate; OC, organic carbon; Pb, lead; Si, silicon; SO_4_^2-^, sulfate; V, vanadium; and Zn, zinc.

Each 1-decile increase in the mixture index was associated with a 4.7% (95% CI, 3.3%-6.3%) increase in the hazard of graft failure after KT. The constituent that contributed the largest weight to the observed associations was SO_4_^2-^, with the relative importance of 51.3%. Detailed distributions of the relative importance of each constituent are shown in the Figure.

Each 1-decile increase in the mixture index was associated with a 3.9% (95% CI, 2.5%-5.2%) increase in the hazard of all-cause mortality among KT recipients within 5 years after KT. Similar to graft failure, the constituents that contributed the largest weights to the observed associations were SO_4_^2-^ (30.1%) and NH_4_^+^ (22.1%). In addition, Pb also contributed, with an estimated importance of 13.3%.

The rankings of the relative importance of each constituent did not change significantly after including the interaction terms in the model (eTables 1-4 in Supplement 1). The exposure-outcome associations were larger for acute rejection, DGF, and DCGF among Black and Hispanic KT recipients and those who belonged to additional racial and ethnic groups compared with those who were White (Table 3). Significant effect measure modification was also observed for CIT in our analysis of the short-term outcomes, and larger associations were observed among those who had longer CIT. Detailed results for the interaction analysis are shown in Table 3. The results of the sensitivity analysis are shown in eTables 5 to 9 in Supplement 1.

**Table 3.** Association of 1-Decile Increase in PM_2.5_ Mixture With Risk of Posttransplant Outcomes Among 192 587 Kidney Transplant Recipients Who Received Transplant in the United States Between 2001 and 2016

Characteristic	Death		Death-censored graft failure		Acute rejection			Delayed graft function	
	aHR (95% CI)	*P *value^a^	aHR (95% CI)	*P *value^a^	aOR (95% CI)	*P *value^a^	aOR (95% CI)		*P *value^a^
Age, y									
<55	1.06 (1.04-1.08)	.04	1.05 (1.03-1.07)	.44	1.03 (1.02-1.05)	.50	1.08 (1.06-1.09)		.02
≥55	1.04 (1.00-1.08)		1.06 (1.02-1.10)		1.03 (0.99-1.07)		1.10 (1.07-1.13)		
Sex									
Male	1.05 (1.04-1.07)	.77	1.05 (1.03-1.07)	.17	1.03 (1.02-1.04)	.06	1.08 (1.07-1.09)		.12
Female	1.05 (1.02-1.08)		1.03 (1.00-1.07)		1.04 (1.02-1.06)		1.09 (1.07-1.12)		
Race									
Other^b^	1.05 (1.03-2.76)	.09	1.08 (1.06-1.09)	.01	1.05 (1.04-1.07)	.008	1.08 (1.07-1.10)		.04
White	1.03 (1.00-1.06)		1.05 (1.02-1.08)		1.01 (0.97-1.06)		1.06 (1.03-1.09)		
Donor type									
Living	1.04 (1.02-1.06)	.64	1.07 (1.04-1.10)	.74	1.01 (1.01-1.02)	<.001	1.13 (1.11-1.16)		<.001
Deceased	1.03 (0.99-1.07)		1.07 (1.02-1.13)		1.05 (1.03-1.06)		1.09 (1.04-1.14)		
CIT									
<12 h	1.05 (1.03-1.06)	.52	1.07 (1.05-1.10)	.13	1.01 (1.01-1.02)	<.001	1.07 (1.05-1.09)		.002
≥12 h	1.04 (1.01-1.07)		1.06 (1.02-1.10)		1.05 (1.03-1.07)		1.10 (1.07-1.14)		

Abbreviations: aHR, adjusted hazard ratio; aOR, adjusted odds ratio; CIT, cold-ischemia time; PM_2.5_, particulate matter with a diameter of 2.5 µm or less.

^a^ *P* for interaction.

^b^ Includes American Indian or Alaska Native, Arab or Middle Eastern, Asian, Black, Hispanic, Indian subcontinent, multiracial, and Native Hawaiian or Other Pacific Islander individuals and those with unknown race and ethnicity.

## Discussion

In this national study among all US adult KT recipients between 2001 and 2016, we observed that higher levels of particulate constituent mixtures were associated with increased risk of adverse post-KT outcomes. For long-term outcomes, SO_4_^2-^ had the highest contribution. Pb was the most important particulate constituent on the associations between particulate constituent mixture and acute rejection. Ni and OC had the highest relative importance in the association between the PM_2.5_ mixture and DGF. To our knowledge, this is the first study to investigate the impact of different PM_2.5_ constituents on post-KT outcomes.

We observed that SO_4_^2-^ had the largest contributions to the association between the particulate constituent mixture and long-term outcomes among KT recipients. The majority of the SO_4_^2-^ in PM_2.5_ comes from coal burning, resulting from the secondary formation of SO_4_^2-^ from SO_2_ emissions. Coal-related PM_2.5_ has been decreasing in the United States during the past decades due to new technology and policy changes, but it remains a large environmental concern in many developing countries.^17,18^ Previous studies^19,20^ found that SO_4_^2-^ in PM_2.5_ was associated with increased risk of multiple diseases, including cancers and infections, suggesting that SO_4_^2-^ could potentially affect the immune system of human body. A panel study^21^ suggested that SO_4_^2-^ could increase oxidative stress and coagulation in human body, which poses great risk to KT recipients. Coagulation could lead to tissue hyperfusion, ischemia, and necrosis, which lead to kidney damage.^22^ Moreover, oxidative stress induced by SO_4_^2-^ particles could also lead to inflammation and potentially immunothrombosis, another important risk factor for graft failure.^15^

More than three-quarters of the observed association of PM_2.5_ mixture with acute rejection was attributable to Pb in our study. The Pb in air pollution significantly decreased after the banning of leaded gasoline in the United States in 1996.^23^ However, Pb continues to be emitted from industrial sources, such as ore and metal processing.^24^ Pb is also higher near roads because of the use of Pb weights to balance tires, which fall off and are ground down by subsequent traffic. Thus, Pb could be serving as a proxy for other unmeasured components of traffic particles. Previous studies^25,26^ suggested that Pb is a heavy metal with potential nephrotoxic effects. Higher exposure to Pb has also been found to be associated with worse long-term outcomes among KT recipients. Sotomayor et al^27^ observed that higher plasma Pb levels were associated with increased risk of graft failure among a cohort of Dutch KT recipients. The observed association of Pb with acute rejection could be due to its effect on immune system. Experimental studies^28,29^ suggested that Pb induced changes in the activity of T cells and decreased the production of interferon-γ, a cytokine which prevents early thrombosis, congestion, and necrosis after KT. Moreover, exposure to Pb could also lead to increased oxidative stress in the system, which is another risk factor for acute rejection.^30,31^

OC and Ni were the top contributors in the association between PM_2.5_ mixtures and DGF. This result suggested that the adverse effect of PM_2.5_ on delayed function is likely to be driven by PM_2.5_ sourced from fuel oil combustion, vehicle emissions, and biomass burning. Environmental risk factors are believed to be less important in the development of DGF.^32^ However, environmental factors could potentially induce some predisposed conditions among KT recipients, which lead to higher vulnerability and thus increase the risk of DGF. In our interaction analysis, we observed that KT recipients with longer CIT experienced greater PM_2.5_ mixture–related risk of DGF, which also suggest that environmental risk factors and transplant factors could potentially interact and lead to worse outcomes among the patients.

Our study found that the primary contributing constituents to the observed association between PM_2.5_ and post-KT outcomes differ depending on the specific outcome. The association between SO_4_^2-^ and long-term outcomes suggested that it may affect KT recipients mainly through a long-term systematic process, such as chronic inflammation.^33^ In contrast, Pb might more directly target immune cell function and antigen presentation.^28,29^ The association of OC and Ni, a potent inducer of oxidative stress, with DGF aligns mechanistically with their potential to cause intense oxidative damage during the critical peritransplant ischemia-reperfusion phase, impairing initial recovery and function.^34,35^ Our results suggested that PM_2.5_ affects KT recipients through multiple mechanisms and that the toxic effects of its constituents vary. PM_2.5_ is currently regulated as a single air pollutant in the United States and many other countries, with most existing studies treating it as a uniform exposure that assumes all constituents and sources of PM_2.5_ have the same toxicity levels.^5,6^ Findings from this study and an increasing body of evidence suggest that the toxic effects and health impacts of PM_2.5_ can vary substantially based on its different constituents and sources.

### Strengths and Limitations

This study has several strengths. First, it included nearly all KT recipients in the United States during the study period, which allowed sufficient power in our analysis and good generalizability of the results. Second, SRTR collects data on a large number of recipient- and donor-related factors, which allowed us to control for important confounders in the study. Moreover, this study used the weighted quantile sum regression to investigate the associations between PM_2.5_ constituents and post-KT outcomes, which allowed us to handle the high correlation between different constituents.

This study is also limited in the following aspects. First, because of confidentiality concerns, we had to use zip code–level exposures instead of individual-level exposures in this study. The actual exposure level could be affected by different individual factors and therefore lead to measurement error. However, a previous study suggested that ambient PM_2.5_ exposure level is a valid proxy for individual exposure and could help avoid some confounding from individual-level confounders.^36^ PM_2.5_ constituent levels could vary over short distances; therefore, the process of aggregating exposures to the zip code level could also lead to measurement error. However, the aggregation process is independent of the outcome status and the measurement of the outcome. Therefore, the measurement is likely to be independent and nondifferential and bias the estimates toward to null. Second, we were only able to obtain zip code information at the year of KT, which may not reflect the real area that the recipient spent most of their time prior to KT. However, the zip code information from SRTR has successfully served as a proxy of physical locations in multiple studies.^6,37^ Third, given that the zip code information was not updated during the study period, we were not able to use a time-varying exposure in this study, which may bias the estimates for long-term outcomes. However, the results remained consistent when we limited the follow-up time to first year after KT. Fourth, although we try to include all the potential confounders that are available to our model, given the observational nature of the study, residual confounding is always possible.

## Conclusions

In this cohort study, PM_2.5_ mixture was associated with increased risk of adverse posttransplant outcomes among KT recipients. Of all the PM_2.5_ constituents included in this study, SO_4_^2-^ had the greatest contribution to long-term outcomes, while Pb, OC, and Ni were more associated with short-term outcomes. KT recipients may benefit from a more targeted PM_2.5_ control strategy than a universal one. Future regulations on PM_2.5_ should consider focusing on the major sources of the PM_2.5_ constituents with larger health impacts. KT recipients and candidates could consider moving to areas with lower levels of harmful constituents or to use HEPA particle filters to optimize posttransplant outcomes.

## Notes

Supplement 1. **eTable 1.** Relative Contributions of PM_2.5 _Constituents in the Association Between the PM_2.5 _Mixture and all-Cause Mortality Across Models With Interaction Terms **eTable 2.** Relative Contributions of PM_2.5 _Constituents in the Association Between the PM_2.5 _Mixture and Death-Censored Graft Failure Across Models With Interaction Terms **eTable 3.** Relative Contributions of PM_2.5 _Constituents in the Association Between the PM_2.5 _Mixture and Acute Rejection Across Models With Interaction Terms **eTable 4.** Relative Contributions of PM_2.5 _Constituents in the Association Between the PM_2.5 _Mixture and Delayed Graft Function Across Models With Interaction Terms **eTable 5.** Relative Contributions of PM_2.5 _Constituents in the Association Between the PM_2.5 _Mixture and Time-to-Event Outcomes (Death Censored Graft Failure and Mortality) During First Year After Kidney Transplant **eTable 6.** Hazard Ratio of Each Decile Increase in PM_2.5 _Mixture on Time-to-Event Outcomes (Death Censored Graft Failure and Mortality) During First Year After Kidney Transplant **eTable 7.** Relative Contributions of PM_2.5 _Constituents in the Association Between the PM_2.5 _Mixture and Post-KT Outcomes When Further Adjusting for NO_2 _and Ozone **eTable 8.** Estimated Effect of Each Decile Increase in PM_2.5 _Mixture on Post-KT Outcomes When Further Adjusting for NO_2 _and Ozone **eFigure.** Correlation Plot Between PM_2.5_ Constituents in the United States

Supplement 2.
Data Sharing Statement
