# Supplementary material for: Particulate Constituents and Posttransplant Outcomes Among Kidney Transplant Recipients
Source: JAMA Netw Open. 2025 Aug 14;8(8):e2527142. doi: 10.1001/jamanetworkopen.2025.27142 (PMC12355287; doi:10.1001/jamanetworkopen.2025.27142)
Supplement: Supplement 1. — eTable 1. Relative Contributions of PM2.5 Constituents in the Association Between the PM2.5 Mixture and all-Cause Mortality Across Models With Interaction Terms eTable 2. Relative Contributions of PM2.5 Constituents in the Association Between the PM2.5 Mixture and Death-Censored Graft Failure Across Models With Interaction Terms eTable 3. Relative Contributions of PM2.5 Constituents in the Association Between the PM2.5 Mixture and Acute Rejection Across Models With Interaction Terms eTable 4. Relative Contributions of PM2.5 Constituents in the Association Between the PM2.5 Mixture and Delayed Graft Function Across Models With Interaction Terms eTable 5. Relative Contributions of PM2.5 Constituents in the Association Between the PM2.5 Mixture and Time-to-Event Outcomes (Death Censored Graft Failure and Mortality) During First Year After Kidney Transplant eTable 6. Hazard Ratio of Each Decile Increase in PM2.5 Mixture on Time-to-Event Outcomes (Death Censored Graft Failure and Mortality) During First Year After Kidney Transplant eTable 7. Relative Contributions of PM2.5 Constituents in the Association Between the PM2.5 Mixture and Post-KT Outcomes When Further Adjusting for NO2 and Ozone eTable 8. Estimated Effect of Each Decile Increase in PM2.5 Mixture on Post-KT Outcomes When Further Adjusting for NO2 and Ozone eFigure. Correlation Plot Between PM2.5 Constituents in the United States [file jamanetwopen-e2527142-s001.pdf]

Supplemental Online Content

Feng Y, Li Y, Bae S, Orandi B, McAdams-Demarco M, Schwartz J. Particulate constituents and posttransplant outcomes among kidney transplant recipients. *JAMA Netw Open.* 2025;8(8):e2527142. doi:10.1001/jamanetworkopen.2025.27142

eTable 1. Relative Contributions of PM2.5 Constituents in the Association Between the PM2.5 Mixture and all-Cause Mortality Across Models With Interaction Terms

eTable 2. Relative Contributions of PM2.5 Constituents in the Association Between the PM2.5 Mixture and Death-Censored Graft Failure Across Models With Interaction Terms

eTable 3. Relative Contributions of PM2.5 Constituents in the Association Between the PM2.5 Mixture and Acute Rejection Across Models With Interaction Terms

eTable 4. Relative Contributions of PM2.5 Constituents in the Association Between the PM2.5 Mixture and Delayed Graft Function Across Models With Interaction Terms

eTable 5. Relative Contributions of PM2.5 Constituents in the Association Between the PM2.5 Mixture and Time-to-Event Outcomes (Death Censored Graft Failure and Mortality) During First Year After Kidney Transplant

eTable 6. Hazard Ratio of Each Decile Increase in PM2.5 Mixture on Time-to-Event Outcomes (Death Censored Graft Failure and Mortality) During First Year After Kidney Transplant

eTable 7. Relative Contributions of PM2.5 Constituents in the Association Between the PM2.5 Mixture and Post-KT Outcomes When Further Adjusting for NO2 and Ozone

eTable 8. Estimated Effect of Each Decile Increase in PM2.5 Mixture on Post-KT Outcomes When Further Adjusting for NO2 and Ozone

This supplemental material has been provided by the authors to give readers additional information about their work.

© 2025 Feng Y et al. *JAMA Network Open*.

eTable 1. Relative Contributions of $PM_{2.5}$ Constituents in the Association Between the $PM_{2.5}$ Mixture and all-Cause Mortality Across Models With Interaction Terms

All-cause mortality									
Age		Sex		Race		Donor type		Cold ischemia time	
Component	weights	Component	weights	Component	weights	Component	weights	Component	weights
so4	0.344	so4	0.269	so4	0.652	nh4	0.329	nh4	0.503
nh4	0.260	nh4	0.197	nh4	0.169	so4	0.312	so4	0.201
k	0.138	v	0.103	no3	0.048	no3	0.149	ni	0.128
v	0.086	ni	0.095	fe	0.041	v	0.055	k	0.040
pb	0.049	no3	0.078	v	0.028	k	0.041	br	0.035
ni	0.043	ec	0.064	pb	0.024	si	0.035	fe	0.026
br	0.024	br	0.063	si	0.023	fe	0.034	no3	0.026
fe	0.022	fe	0.062	k	0.007	br	0.014	ec	0.016
no3	0.019	k	0.027	ca	0.003	pb	0.012	si	0.010
si	0.009	si	0.009	ec	0.003	ni	0.011	oc	0.005
ca	0.003	z	0.008	br	0.001	ca	0.007	v	0.005
ec	0.002	oc	0.008	cu	0.001	zn	0.000	cu	0.003
oc	0.000	ca	0.007	ni	0.001	oc	0.000	pb	0.001
cu	0.000	pb	0.006	zn	0.000	cu	0.000	ca	0.001
zn	0.000	cu	0.005	oc	0.000	ec	0.000	zn	0.000

© 2025 Feng Y et al. *JAMA Network Open*.

eTable 2. Relative Contributions of $PM_{2.5}$ Constituents in the Association Between the $PM_{2.5}$ Mixture and Death-Censored Graft Failure Across Models With Interaction Terms

Death-censored graft failure									
Age		Sex		Race		Donor type		Cold ischemia time	
Component	weights	Component	weights	Component	weights	Component	weights	Component	weights
so4	0.579	so4	0.409	so4	0.753	so4	0.323	so4	0.462
ca	0.122	nh4	0.299	si	0.131	si	0.254	si	0.162
si	0.104	si	0.073	v	0.076	v	0.140	v	0.125
fe	0.047	br	0.060	k	0.013	pb	0.119	nh4	0.088
ni	0.036	ca	0.039	pb	0.009	nh4	0.060	ni	0.067
pb	0.035	ni	0.035	ni	0.007	fe	0.038	ca	0.058
k	0.034	fe	0.028	ca	0.003	ca	0.035	pb	0.014
br	0.014	v	0.025	nh4	0.003	k	0.017	br	0.011
v	0.008	ec	0.013	fe	0.002	ec	0.005	fe	0.006
nh4	0.007	cu	0.007	cu	0.001	cu	0.004	ec	0.003
cu	0.006	pb	0.006	br	0.001	ni	0.002	k	0.002
no3	0.004	no3	0.005	no3	0.001	no3	0.002	cu	0.001
zn	0.001	k	0.001	ec	0.000	zn	0.001	no3	0.000
oc	0.001	zn	0.000	zn	0.000	oc	0.000	zn	0.000
ec	0.001	oc	0.000	oc	0.000	br	0.000	oc	0.000

© 2025 Feng Y et al. *JAMA Network Open*.

eTable 3. Relative Contributions of $PM_{2.5}$ Constituents in the Association Between the $PM_{2.5}$ Mixture and Acute Rejection Across Models With Interaction Terms

Acute rejection									
Age		Sex		Race		Donor type		Cold ischemia time	
Component	weights	Component	weights	Component	weights	Component	weights	Component	weights
pb	0.400	pb	0.591	pb	0.362	pb	0.638	pb	0.612
si	0.163	so4	0.124	so4	0.281	zn	0.117	so4	0.112
so4	0.143	si	0.082	nh4	0.206	so4	0.102	nh4	0.086
k	0.087	br	0.073	zn	0.092	nh4	0.058	zn	0.046
nh4	0.059	nh4	0.038	k	0.035	si	0.031	br	0.041
zn	0.049	zn	0.037	br	0.012	k	0.017	si	0.041
ca	0.044	k	0.036	si	0.010	ca	0.013	fe	0.017
br	0.042	fe	0.007	ca	0.001	br	0.011	k	0.016
fe	0.005	ni	0.006	ec	0.001	ni	0.009	ni	0.014
no3	0.004	ca	0.004	fe	0.000	fe	0.002	ec	0.010
ni	0.003	ec	0.003	ni	0.000	ec	0.001	ca	0.005
ec	0.001	oc	0.000	no3	0.000	oc	0.001	oc	0.000
oc	0.000	v	0.000	oc	0.000	no3	0.000	cu	0.000
cu	0.000	cu	0.000	v	0.000	cu	0.000	no3	0.000
v	0.000	no3	0.000	cu	0.000	v	0.000	v	0.000

© 2025 Feng Y et al. *JAMA Network Open*.

eTable 4. Relative Contributions of $PM_{2.5}$ Constituents in the Association Between the $PM_{2.5}$ Mixture and Delayed Graft Function Across Models With Interaction Terms

Delayed graft function									
Age		Sex		Race		Donor type		Cold ischemia time	
Component	weights	Component	weights	Component	weights	Component	weights	Component	weights
oc	0.538	oc	0.420	oc	0.378	oc	0.471	oc	0.359
ni	0.282	ni	0.330	ni	0.174	v	0.193	ni	0.241
cu	0.111	cu	0.150	no3	0.173	ni	0.188	cu	0.214
v	0.064	br	0.088	br	0.132	cu	0.126	v	0.148
ec	0.002	v	0.008	ec	0.075	br	0.020	br	0.037
br	0.001	no3	0.002	zn	0.061	no3	0.002	ec	0.001
si	0.000	zn	0.001	nh4	0.003	ec	0.000	no3	0.000
no3	0.000	fe	0.001	cu	0.002	nh4	0.000	si	0.000
nh4	0.000	k	0.000	si	0.001	ca	0.000	ca	0.000
pb	0.000	nh4	0.000	pb	0.000	si	0.000	fe	0.000
zn	0.000	si	0.000	v	0.000	fe	0.000	pb	0.000
fe	0.000	so4	0.000	fe	0.000	k	0.000	k	0.000
ca	0.000	ca	0.000	ca	0.000	so4	0.000	so4	0.000
k	0.000	ec	0.000	so4	0.000	pb	0.000	zn	0.000
so4	0.000	pb	0.000	k	0.000	zn	0.000	nh4	0.000

© 2025 Feng Y et al. *JAMA Network Open*.

eTable 5. Relative Contributions of $PM_{2.5}$ Constituents in the Association Between the $PM_{2.5}$ Mixture and Time-to-Event Outcomes (Death Censored Graft Failure and Mortality) During First Year After Kidney Transplant

Death		Death censored graft-failure	
Component	weights	Component	weights
so4	0.403	so4	0.482
si	0.225	v	0.169
v	0.165	si	0.113
fe	0.067	pb	0.077
pb	0.064	nh4	0.061
k	0.023	ni	0.053
oc	0.016	oc	0.021
cu	0.014	k	0.013
no3	0.008	ca	0.009
ca	0.003	fe	0.001
ec	0.003	ec	0.001
ni	0.003	cu	0.000
z	0.002	no3	0.000
nh4	0.002	br	0.000
br	0.001	z	0.000

© 2025 Feng Y et al. *JAMA Network Open*.

eTable 6. Hazard Ratio of Each Decile Increase in $PM_{2.5}$ Mixture on Time-to-Event Outcomes (Death Censored Graft Failure and Mortality) During First Year After Kidney Transplant

	aHR (95%CI)
Death	1.137 (1.111, 1.164)
Death censored graft failure	1.092 (1.070, 1.113)

© 2025 Feng Y et al. *JAMA Network Open*.

eTable 7. Relative Contributions of $PM_{2.5}$ Constituents in the Association Between the $PM_{2.5}$ Mixture and Post-KT Outcomes When Further Adjusting for NO_2 and Ozone

Acute rejection		Delayed graft function		DCGF		Death	
Component	Weights	Component	Weights	Component	Weights	Component	Weights
pb	0.308	ni	0.436	so4	0.364	so4	0.246
z	0.171	oc	0.401	v	0.132	nh4	0.171
si	0.116	v	0.097	si	0.082	ni	0.095
so4	0.101	cu	0.059	pb	0.067	no3	0.089
br	0.096	br	0.007	nh4	0.066	v	0.088
k	0.073	no3	0	z	0.053	si	0.065
nh4	0.062	z	0	ec	0.04	pb	0.056
ca	0.039	nh4	0	ca	0.04	br	0.046
fe	0.02	ec	0	br	0.039	ec	0.038
ni	0.011	ca	0	cu	0.034	ca	0.035
ec	0.001	si	0	ni	0.031	k	0.034
cu	0	pb	0	no3	0.03	oc	0.014
oc	0	fe	0	fe	0.014	z	0.009
no3	0	k	0	k	0.007	fe	0.008
v	0	so4	0	oc	0.002	cu	0.007

DCGF: Death-censored graft failure

© 2025 Feng Y et al. *JAMA Network Open*.

eTable 8. Estimated Effect of Each Decile Increase in $PM_{2.5}$ Mixture on Post-KT Outcomes When Further Adjusting for NO_2 and Ozone

	Effect estimates (95%CI)
Acute rejection	1.051 (1.030, 1.074)
Delayed graft function	1.086 (1.072, 1.100)
Death	1.051 (1.035, 1.067)
DCGF	1.065 (1.033, 1.099)

DCGF: Death censored graft failure

For acute rejection and delayed graft failure, the effect estimates are odds ratio while for Death and DCGF, the effect estimates were hazard ratios

© 2025 Feng Y et al. *JAMA Network Open*.

Table S9. Association between total PM2.5 and graft failure (treating death as censoring VS treating death as competing event)

	HR
Cox model	1.013 (1.007, 1.019)
Fine and Gray's model	1.012 (1.006, 1.017)

HR: hazard ratio for each 1μg/m^3 increase in PM2.5; Cox model estimated the association between PM2.5 and death-censored graft failure; Fine and Gray's estimated the association between PM2.5 and graft failure while treating death as a competing event.

© 2025 Feng Y et al. *JAMA Network Open*.

eFigure. Correlation Plot Between $PM_{2.5}$ Constituents in the United States

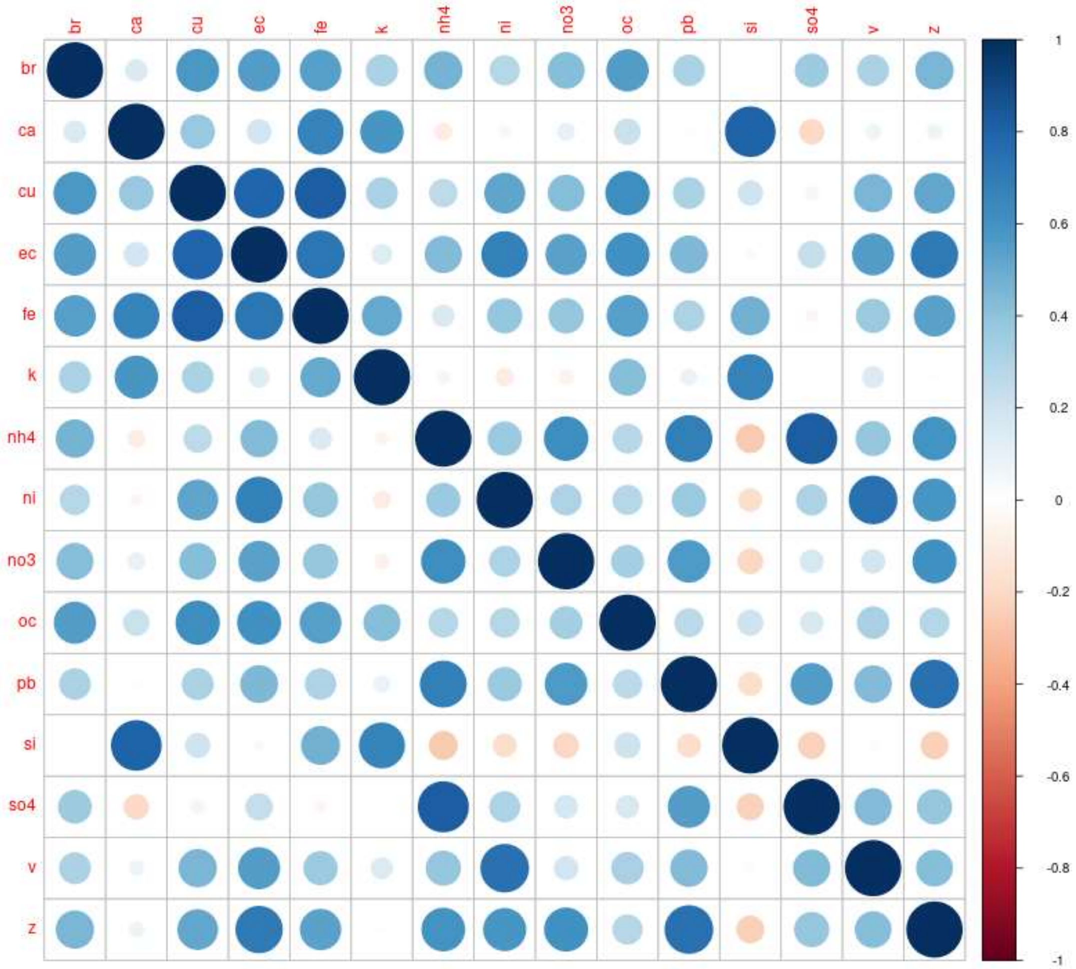

© 2025 Feng Y et al. *JAMA Network Open*.
