# Supplementary material for: Particulate Constituents and Posttransplant Outcomes Among Kidney Transplant Recipients
Source: JAMA Netw Open. 2025 Aug 14;8(8):e2527142. doi: 10.1001/jamanetworkopen.2025.27142 (PMC12355287; doi:10.1001/jamanetworkopen.2025.27142)
Supplement: Supplement 2. — Data Sharing Statement [file jamanetwopen-e2527142-s002.pdf]

Data Sharing Statement

Feng. Particulate Constituents and Posttransplant Outcomes Among Kidney Transplant Recipients. *JAMA Netw Open*. Published August 14, 2025.
doi:10.1001/jamanetworkopen.2025.27142

Data
Data available: No
